# U-Shaped Relation of Dietary Thiamine Intake and New-Onset Hypertension

**DOI:** 10.3390/nu14163251

**Published:** 2022-08-09

**Authors:** Yuanyuan Zhang, Yanjun Zhang, Sisi Yang, Ziliang Ye, Qimeng Wu, Mengyi Liu, Chun Zhou, Panpan He, Jianping Jiang, Min Liang, Guobao Wang, Fanfan Hou, Chengzhang Liu, Xianhui Qin

**Affiliations:** 1 Division of Nephrology, National Clinical Research Center for Kidney Disease, State Key Laboratory of Organ Failure Research, Guangdong Provincial Institute of Nephrology, Guangdong Provincial Key Laboratory of Renal Failure Research, Nanfang Hospital, Southern Medical University, Guangzhou 510515, China; 2 Department of Epidemiology and Biostatistics, School of Public Health, Anhui Medical University, Hefei 230032, China; 3 Institute of Biomedicine, Anhui Medical University, Hefei 230032, China

**Keywords:** dietary thiamine intake, nationwide cohort study, new-onset hypertension

## Abstract

Background: To examine the relation of dietary thiamine intake with risk of new-onset hypertension in the general adults. Methods: A total of 12,177 participants without hypertension at baseline from China Health and Nutrition Survey (CHNS) were included. The study outcome was new-onset hypertension, which was defined as a systolic blood pressure ≥140 mm Hg or a diastolic blood pressure ≥90 mm Hg or under antihypertensive treatment or diagnosed by physician during the follow-up. Results: A total of 4269 participants occurred new-onset hypertension over a median follow-up of 6.1 years. Overall, there was a U-shaped relation (*p* for nonlinearity <0.001) of dietary thiamine intake with new-onset hypertension, with an inflection point at 0.93 mg/day. Accordingly, in the threshold effect analysis, there was an inverse association between dietary thiamine intake (per SD increment: HR, 0.62; 95% CI: 0.53, 0.72) and new-onset hypertension in participants with dietary thiamine intake <0.93 mg/day, and a positive association between dietary thiamine intake (per SD increment: HR, 1.38; 95% CI: 1.32, 1.44) and new-onset hypertension in those with dietary thiamine intake ≥0.93 mg/day. Conclusion: The association between dietary thiamine intake and the risk of new-onset hypertension followed a U-shaped relation in the general Chinese population, with an inflection point at 0.93 mg/day of dietary thiamine intake.

## 1. Introduction

Hypertension is one of the major risk factors for cardiovascular disease (CVD) [1,2]. The global age-standardized prevalence of hypertension was 20.1% in women and 24.1% in men in 2015 [3]. Therefore, identifying more modifiable risk factors of hypertension, such as diets and lifestyles [4], is of great clinical implication for primary prevention of hypertension and its related complications.

Thiamine, also referred to as vitamin B1, is a water-soluble vitamin [5]. Thiamine has important roles in the energy metabolism, anti-inflammation, endothelial function and oxidative stress [5,6,7]. Accordingly, an animal experiment showed that thiamine repletion could reduce blood pressure (BP) in experimental hypertensive rats [8]. However, little research has examined the association between dietary thiamine intake and new-onset hypertension. Therefore, to date, the prospective relation of dietary thiamine intake with new-onset hypertension is still unknown.

To address the above gaps in knowledge, based on data from the China Health and Nutrition Survey (CHNS), we aimed to investigate the association of dietary thiamine intake with risk of hypertension in the general adults.

## 2. Methods

### 2.1. Study Design and Population

Our current analyses were based on the CHNS. CHNS is an ongoing, national, multipurpose, open, prospective study initiated in 1989. CHSN has been followed up in 1991, 1993, 1997, 2000, 2004, 2006, 2009, 2011 and 2015. Details of the study design and some results of the CHNS have been published elsewhere [9,10,11,12,13,14]. 

In our present study, seven rounds of CHNS data from 1997 to 2015 were included. First, participants who were <18 years of age, pregnant or having no information on BP data at the time of the survey were excluded. Then, those who were surveyed at only one round were excluded. Furthermore, participants having hypertension or having no dietary thiamine data or having implausible energy intake data (female: >3600 or <500 kcal/day; male: >4200 or <600 kcal/day) [15] were also excluded. Finally, we included 12,177 participants in the current analyses (Supplemental Figure S1).

The study was approved by the Institutional Review committees of the National Institute for Nutrition and Health, Chinese Center for Disease Control and Prevention (No. 201524) and the Institutional Review committees of the University of North Carolina at Chapel Hill. All participants gave written, informed consent.

### 2.2. Dietary Nutrients Intake Assessments

In the CHNS, through a face-to-face interview, trained nutritionists collected both individual and household level dietary data in each survey round. Three consecutive 24 h dietary recalls and a household food inventory were used to collect dietary intake. The accuracy of 24 h dietary recalls has been validated [16,17]. We converted food consumption data into the intakes of nutrients and total energy according to the Chinese Food Composition Table (FCT). In the current analyses, 3-day average intakes of dietary macronutrients and micronutrients at each round were used. 

Furthermore, we calculated the cumulative average intake values of each nutrient, using results from baseline to last visit prior to the date of new-onset hypertension or all results among those without new-onset hypertension, to minimize the within-person variation and represent long-term status of dietary intake. 

### 2.3. Blood Pressure and Covariates Measurements

In each survey round, after the participants had rested for 5 min, with appropriately sized cuffs and a standard method, seated BP was measured using a mercury manometer. Three independent measurements were taken on the same arm. In the current analyses, we calculated the mean diastolic BP (DBP) and systolic BP (SBP) of the three measurements.

Lifestyle and demographic information were collected through questionnaires, including sex, age, urban or rural residents, occupations, regions, education levels and smoking and alcohol drinking statuses. Body mass index (BMI) was a ratio of weight (kg) in comparison to height squared (m^2^).

### 2.4. Study Outcome

New-onset hypertension, defined as an SBP ≥140 mm Hg or a DBP ≥90 mm Hg or under antihypertensive treatment or diagnosed by physician during the follow-up, was the study outcome. 

### 2.5. Statistical Analysis

Characteristics of the participants, shown as proportions for categorical variables and means ± standard deviations (SDs) for continuous variables according to quartiles of dietary thiamine intake (<0.76, 0.76–< 0.93, 0.93–< 1.13, ≥1.13 mg/day), were compared using chi-square tests for categorical variables or analysis of variance (ANOVA) for continuous variables, accordingly. 

The study baseline was defined as the year in which each participant first participated in the survey. The follow-up person-year for each participant was calculated from baseline to the time of the first diagnosis of hypertension (the middle date between the first diagnostic survey and the most recent survey before), the last round of the survey before withdrawal from the study or the end of the latest survey (2015), whichever came first. A 2-piecewise Cox regression analysis was performed to estimate the threshold effect of the dietary thiamine intake on the risk of hypertension using a smoothing function. The inflection point was determined by likelihood ratio tests and bootstrap resampling methods. The relation of dietary thiamine intake with new-onset hypertension was examined using Cox proportional hazards models without and with adjustments for sex, age, BMI, survey year, SBP, DBP, smoking, alcohol drinking, urban or rural residents, education levels, regions, occupations, physical activity levels, energy intake, sodium intake and potassium intake. We assessed the significance of the interaction between exposures and log-transformed follow-up time to test the proportional hazards assumption and found no clear evidence of violation. In addition, we performed restricted cubic spline (RCS) Cox regression, with 4 knots (20th, 40th, 60th, 80th percentiles of dietary intake of thiamine), to evaluate the shape of dose–response association of dietary thiamine intake with new-onset hypertension. Furthermore, stratified analyses and interaction testing were conducted to evaluate the possible modifiers on the relation of dietary thiamine intake with new-onset hypertension. 

All analyses were two-tailed, and a *p* value less than 0.05 was deemed as statistically significant. R software, version 3.6.3 (R Foundation for Statistical Computing, Vienna, Austria, http://www.R-project.org, accessed on 17 June 2020) was used in data analyses. 

## 3. Results

### 3.1. Study Population and Characteristics

The flow chart of current study is illustrated in Supplemental Figure S1. A total of 12,177 participants were included in the present study. The mean age of the participants was 41.2 ± 14.2 years, and 46.8% were male. The median dietary thiamine intake was 0.93 (25th–75th, 0.76–1.13) mg/day. Characteristics of study participants are shown by quartiles of dietary thiamine intake in Table 1. Participants who had higher dietary thiamine intake were younger, more likely to be rural residents, male, farmers, alcohol drinkers and smokers and had lower education levels, higher physical activity levels and higher intakes of energy, protein, carbohydrate, fat, potassium and sodium (Table 1). 

### 3.2. Association of Dietary Thiamine Intake with New-Onset Hypertension

A total of 4269 (44.9 per 1000 person-years) participants occurred new-onset hypertension over a median follow-up period of 6.1 years (25th–75th, 3.6–11.4 years). Of these, 826 (19.3%) were diagnosed with hypertension by a physician, 526 (12.3%) reported taking antihypertensive drugs and 3923 (91.9%) had an SBP ≥140 mmHg or a DBP ≥90 mmHg during the follow-up. Some patients met at least two of the three criteria. 

**Figure 1:**
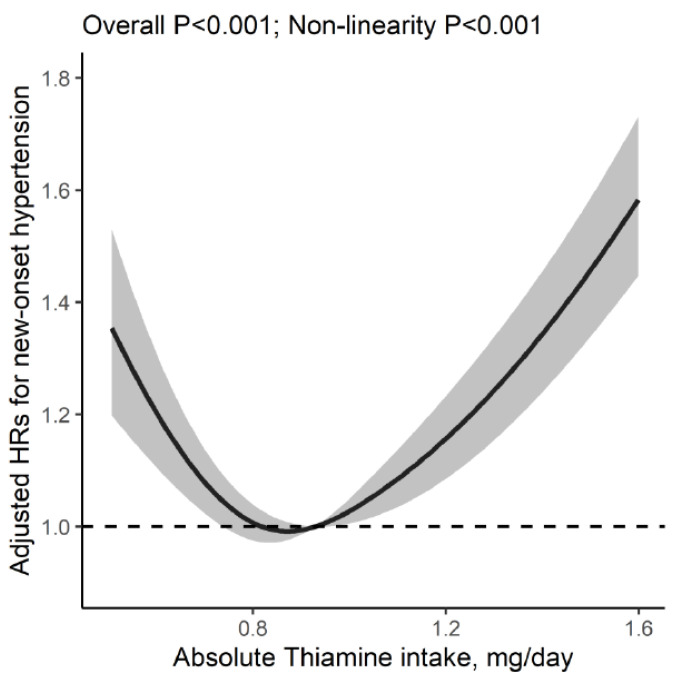
Association between dietary thiamine intake and new-onset hypertension. Adjusted for sex, age, body mass index, survey year, regions, SBP, DBP, alcohol drinking, smoking, urban or rural residents, education levels, occupations, physical activity, intakes of energy, sodium and potassium. Grey area indicates the 95% confidence interval.

We found a U-shaped relation of dietary thiamine intake with new-onset hypertension (*p* for nonlinearity <0.001), with an inflection point at 0.93 mg/day (Figure 1). Accordingly, the threshold effect analysis showed that there was an inverse association between dietary thiamine intake (per SD increment: HR, 0.62; 95% CI: 0.53,0.72) and new-onset hypertension in participants with dietary thiamine intake <0.93 mg/day, and a positive association between dietary thiamine intake (per SD increment: HR, 1.38; 95% CI: 1.32, 1.44) and new-onset hypertension in those with dietary thiamine intake ≥0.93 mg/day. (Table 2). When dietary thiamine intake was assessed as quartiles, compared with participants in the second and third quartiles (0.76–1.13 mg/day, including the inflection point: 0.93 mg/day), the risk of new-onset hypertension was higher not only in participants in the first quartile (<0.76 mg/day; adjusted HR, 1.25; 95% CI, 1.14, 1.37) but in those in the fourth quartile (≥1.13 mg/day; adjusted HR, 1.36; 95% CI, 1.25, 1.47) (Table 3). Moreover, we found similar results for the three different components of new-onset hypertension (Table 4). 

To examine the robustness of the results, the sensitivity analyses were performed. First, further adjustments for intake of other nutrients that have been found to affect new-onset hypertension, including vitamin A [13], riboflavin [10], niacin [12], copper [18] or zinc (Supplemental Table S1), or the intakes of the major food sources of dietary thiamine, including nuts, whole grain, refined grain, vegetables and fruits (Supplemental Table S2), did not substantially alter the results. In addition, we assessed the association between dietary thiamine intake and new-onset hypertension in men and women, respectively. As shown in Supplemental Figure S2, we found a U-shaped relation of dietary thiamine intake with new-onset hypertension in both men and women.

### 3.3. Stratified Analyses

**Figure 2:**
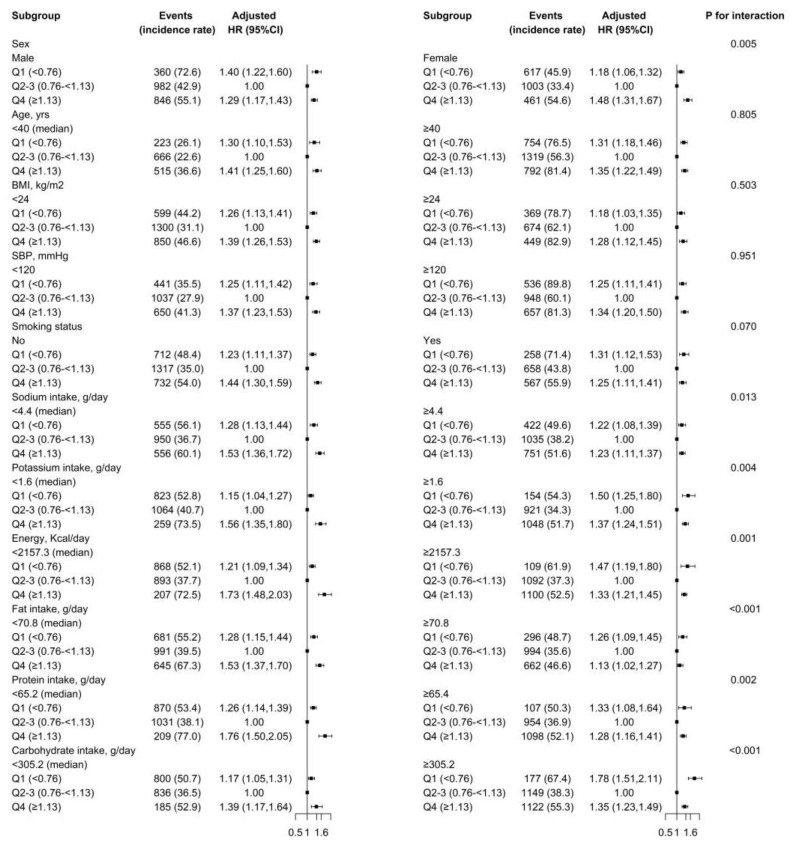
The relation of dietary thiamine intake (Q1 vs. Q2–3 vs. Q4) with risk of new-onset hypertension in various subgroups. Adjusted for sex, age, body mass index, survey year, regions, SBP, DBP, alcohol drinking, smoking, urban or rural residents, education levels, occupations, physical activity, intakes of energy, sodium and potassium, if not stratified.

Stratified analyses were further conducted to evaluate the association of dietary thiamine (≥1.13 vs. 0.76–<1.13 vs. <0.76 mg/day) and new-onset hypertension in various subgroups (Figure 2). There was a U-shaped association between dietary thiamine intake with new-onset hypertension in all subgroups.

None of the variables, including age (median, <40 vs. ≥40 years), BMI (<24 vs. ≥24 kg/m^2^), SBP (<120 vs. ≥120 mmHg) or smoking (no vs. yes), significantly modified the association between dietary thiamine intake (≥1.13 vs. 0.76–<1.13 vs. <0.76 mg/day) and new-onset hypertension (Figure 2). Though the *p* values for the interactions for sex (males vs. females), sodium intake (median, <4.4 vs. ≥4.4 g/d), potassium intake (median, <1.6 vs. ≥1.6 g/d), energy intake (median, <2157.3 vs. ≥2157.3 kcal/d), fat intake (median, <70.8 vs. ≥70.8 g/d), protein intake (median, <65.2 vs. ≥65.2 g/d) and carbohydrate intake (median, <305.1 vs. ≥305.1 g/d) were <0.05, the findings may not have obvious clinical implications considering the similar directionality of the relations and the multiple testing.

## 4. Discussion

In this national, longitudinal cohort study in general Chinese adults, we first demonstrated a U-shaped relation of dietary thiamine intake with new-onset hypertension, with an inflection point at 0.93 mg/day and the lower risk of new-onset hypertension at 0.76–1.13 mg/day of dietary thiamine intake. 

A previous randomized trial of 12 hyperglycemic subjects showed that high-dose thiamine supplementation (3 × 100 mg/day versus placebo) for six weeks was associated with decreased DBP levels [19]. However, Wilkinson TJ et al. found that a marginally significant decrease in SBP (*p* = 0.05) associated with oral thiamine for 3 months was only observed in subjects (> or = 65 y) with persistently low erythrocyte thiamine pyrophosphate concentrations (*n* = 35), and no obvious effect was found in subjects without persistently low RBC thiamine concentrations (*n* = 41) [20]. Of note, these randomized trials mainly examined the effects of short-term (6–12 weeks), high-dose extra thiamine supplementation rather than the long-term effects of dietary thiamine derived from foods in the general population. To date, the prospective relation of dietary thiamine intake with new-onset hypertension remains uncertain. Our present study used a prospective design with a relatively large sample size, which may increase the accuracy and reliability of the effect estimates, and provided an opportunity to explore the dose–response relationship of dietary thiamine intake with new-onset hypertension in a nationwide cohort in China. 

Overall, there was a U-shaped relation of dietary thiamine intake with new-onset hypertension. First, the risk of new-onset hypertension significantly decreased with the increment of dietary thiamine intake in participants with dietary thiamine intake <0.93 mg/day. Some possible mechanisms supported our findings. Endothelial dysfunction is considered to be the initial stage in the development of arterial hypertension and atherosclerosis [21]. It is reported that endothelial function could be improved in a thiamine-rich environment [22]. Moreover, animal models have shown that benfotiamine reduced oxidative stress and activated endothelial nitric oxide synthase to enhance the generation and bioavailability of nitric oxide (NO), subsequently improving the integrity of vascular endothelium and preventing vascular endothelial dysfunction (VED) [23]. In addition, thiamin could play a protective role on macrophages by suppressing the oxidative stress–induced activation of NF-κB (necrosis factor), which induces macrophages to release a variety of inflammatory markers such as cytokines, chemokines, growth factors and immune-responsive proteins [24]. Based upon this evidence, we speculated that the possible biological explanation by which thiamine reduces the risk of hypertension may involve improved endothelial function and antioxidant and anti-inflammatory effects. However, further studies should be conducted to explore the detailed mechanisms. 

Second, among participants with dietary thiamine intake ≥0.93 mg/day, the risk of new-onset hypertension significantly increased with the increment of dietary thiamine intake. To date, there is a lack of mechanisms of toxicity from high thiamin intake from food or supplements. However, Food and Nutrition Board (FNB) has concluded that excessive consumption of thiamin might cause adverse effects [25]. Accordingly, a recent meta-analysis suggested that thiamine supplementation alone increased the risk of mortality in critically ill patients [26]. Furthermore, it is reported that thiamine supplementation could stimulate tumor proliferation [27]. In addition, high thiamine supplementation was related to increased appetite, energy intake and body weight [28]. Of note, the requirement for thiamine is considerably increased by intense exertion and by the onset of wasting disease. On the other hand, the intake of lipids slightly decreases the requirement. The body can only store a small amount of thiamine, and the absorption efficiency is not high. Moreover, the absorption efficiency may be even lower when the gastrointestinal tract is weakened, such as in progressive lethargy, in which case, thiamine can be excreted quickly. Therefore, thiamine overdosage is usually not a problem. However, our study showed that the intake levels of dietary thiamine associated with higher risk of new-onset hypertension is in the relatively low range, underscoring the importance of an optimal thiamine intake level for prevention of new-onset hypertension. Moreover, foods that are high in thiamine may also be high in other nutrients or dietary factors which may influence risk of hypertension. Although sodium, potassium and total energy, as well as vitamin A, riboflavin, niacin, copper and zinc, were included in our regression model, we could not exclude the possibility that other dietary factors might be influencing risk of hypertension. Overall, future research is needed to confirm our findings and further explore the underlying mechanisms. 

Of note, the optimal range of thiamine (0.76–1.13 mg/day) in our study is consistent with the Recommended Dietary Allowance (RDAs) of thiamine (0.78–1.13 mg/day) for adults established by European Food Safety Authority (EFSA) [29]. However, the thiamine RDAs established by US Institute of Medicine (IOM) for men and women were 1.2 mg/day and 1.1 mg/day, respectively [30], which is different from our results. The possible explanations are as follows: the thiamine RDAs established by IOM were mainly based on the controlled depletion–repletion study by Sauberlich [31], though the energy intake (2800 to 3600 kcal/day) in that study [31] was much higher than that in our study. Additionally, the body size of US population is larger than that of the Chinese. 

Nuts, whole grains, vegetables, fruits, meats and fish are all rich in thiamine and among the good food sources of thiamine [25]. Nuts, whole grains, vegetables and fruits are all included in Mediterranean diet and the Dietary Approaches to Stop Hypertension (DASH) diet. Therefore, both the Mediterranean diet and DASH diet should be good dietary patterns for dietary thiamine intake. In addition, the Japanese diet contains vegetables, meat and fish and thus could also provide adequate thiamine intake.

Some limitations of our study should also be noted. First, limiting of observational study, unmeasured and residual confounding remains possible, although we had adjusted a number of dietary foods, nutrients and a series of traditional covariates in the analysis. Moreover, in the related China FCTs, no detailed information was provided for vitamin B6, B9 and B12, which were implicated in the production of different cytokines (including thiamine) or the reduction of homocysteine [32]. As such, we were unable to assess whether these nutrients could affect our findings. Second, the CHNS did not provide information for blood thiamine concentration. Therefore, we could not investigate the association between blood thiamine and the risk of hypertension. However, serum thiamine levels can be affected by a variety of factors and are short-lived. Meanwhile, thiamine levels in the blood account for only 0.8% of the body’s thiamine. As such, serum thiamine is not a reliable indicator of total body thiamine stores [7]. Third, our study was conducted in the general Chinese population; whether the findings can be extrapolated to other populations needs further investigation. Further studies to confirm our results are necessary. 

## 5. Conclusions

In conclusion, we first found a U-shaped relation of dietary thiamine intake with the risk of new-onset hypertension in the general Chinese population, with an inflection point at 0.93 mg/day and lower risk of new-onset hypertension at 0.76–1.13 mg/day of dietary thiamine intake. If further confirmed, our results suggest the importance of maintaining an optimal thiamine intake level for primary prevention of hypertension.

## Figures and Tables

**Table 1:** Characteristics of the participants by quartiles of dietary thiamine intake ^*^.

Variables	Thiamine Intake by Quartiles, mg/day				*p* Value
	Q1 (<0.76)	Q2 (0.76–<0.93)	Q3 (0.93–<1.13)	Q4 (≥1.13)	
N	3044	3044	3044	3045	
Male, No. (%)	948 (31.1)	1258 (41.3)	1531 (50.3)	1961 (64.4)	<0.001
Age, years	44.7 ± 16.0	41.1 ± 13.7	39.5 ± 13.1	39.5 ± 13.2	<0.001
Body mass index, kg/m^2^	22.5 ± 3.3	22.3 ± 3.0	22.3 ± 3.0	22.4 ± 2.9	0.029
Systolic blood pressure, mmHg	114.6 ± 11.9	113.0 ± 11.5	113.3 ± 11.3	114.6 ± 10.9	<0.001
Diastolic blood pressure, mmHg	74.1 ± 8.0	73.8 ± 7.9	74.1 ± 7.8	74.7 ± 7.6	<0.001
Physical activity, MET-hours/week	125.0 ± 91.6	144.2 ± 92.9	149.1 ± 91.4	151.9 ± 95.8	<0.001
Smoking, No. (%)	657 (21.7)	808 (26.6)	980 (32.4)	1243 (41.0)	<0.001
Alcohol drinking, No. (%)	760 (25.1)	908 (30.2)	1086 (36.2)	1384 (45.9)	<0.001
Urban residence, No. (%)	1389 (45.6)	1105 (36.3)	1010 (33.2)	901 (29.6)	<0.001
Regions, No. (%)					<0.001
Central	1373 (45.1)	1189 (39.1)	1249 (41.0)	1771 (58.2)	
North	870 (28.6)	655 (21.5)	545 (17.9)	414 (13.6)	
South	801 (26.3)	1200 (39.4)	1250 (41.1)	860 (28.2)	
Occupation, No. (%)					<0.001
Farmer	702 (23.3)	1105 (36.7)	1192 (39.6)	1349 (44.8)	
Worker	315 (10.4)	386 (12.8)	373 (12.4)	383 (12.7)	
Retire	1069 (35.4)	753 (25.0)	644 (21.4)	571 (19.0)	
Other	932 (30.9)	764 (25.4)	803 (26.7)	706 (23.5)	
Education, No. (%)					<0.001
Illiteracy	640 (21.4)	559 (18.8)	501 (16.7)	505 (16.9)	
Primary school	499 (16.7)	600 (20.2)	638 (21.3)	590 (19.8)	
Middle school	859 (28.7)	973 (32.7)	1067 (35.6)	1092 (36.6)	
High school or above	995 (33.2)	840 (28.3)	793 (26.4)	793 (26.6)	
Dietary intake					
Thiamine, mg/day	0.6 ± 0.1	0.8 ± 0.0	1.0 ± 0.1	1.4 ± 0.3	<0.001
Energy, Kcal/day	1684.1 ± 374.9	2082.6 ± 330.1	2315.9 ± 354.1	2629.7 ± 468.7	<0.001
Fat, g/day	65.0 ± 26.9	72.5 ± 25.7	78.8 ± 27.6	80.9 ± 34.3	<0.001
Protein, g/day	50.7 ± 13.7	62.7 ± 12.6	70.6 ± 13.0	83.3 ± 18.1	<0.001
Carbohydrate, g/day	224.0 ± 70.9	294.8 ± 67.3	331.0 ± 72.9	392.1 ± 99.6	<0.001
Sodium, g/day	4.8 ± 3.1	4.8 ± 2.8	5.1 ± 3.0	5.4 ± 3.1	<0.001
Potassium, g/day	1.3 ± 0.4	1.6 ± 0.4	1.7 ± 0.5	2.1 ± 0.7	<0.001

* The continuous variables were presented as Means ± SDs, and the categorical variables were presented as n (%).

**Table 2:** Threshold analyses of dietary thiamine intake (per SD increment, 0.35 mg/day) on new-onset hypertension using 2-piecewise regression models.

Thiamine Intake, mg/day	Crude Model		Thiamine Intake, mg/day	Adjusted Model *	
	HR (95% CI)	*p* Value		HR (95% CI)	*p* Value
<0.89	0.57 (0.50,0.64)	<0.001	<0.93	0.62 (0.53,0.72)	<0.001
≥0.89	1.31 (1.27,1.36)	<0.001	≥0.93	1.38 (1.32,1.44)	<0.001

* Adjusted for sex, age, body mass index, survey year, regions, SBP, DBP, alcohol drinking, smoking, urban or rural residents, education levels, occupations, physical activity, intakes of energy, sodium and potassium.

**Table 3:** Association between dietary thiamine intake and new-onset hypertension.

Thiamine Intake, mg/day	N	Cases (Incidence Rate ^†^)	Crude Model		Adjusted Model *	
			HR (95% CI)	*p* Value	HR (95% CI)	*p* Value
Quartiles						
Q1 (<0.76)	3044	977 (53.1)	ref		ref	
Q2 (0.76–<0.93)	3044	981 (38.3)	0.70 (0.64, 0.77)	<0.001	0.82 (0.74, 0.90)	<0.001
Q3 (0.93–<1.13)	3044	1004 (36.8)	0.67 (0.62, 0.74)	<0.001	0.78 (0.70, 0.87)	<0.001
Q4 (≥1.13)	3045	1307 (54.9)	1.02 (0.94, 1.10)	0.702	1.08 (0.95, 1.22)	0.229
Categories						
Q1 (<0.76)	3044	977 (53.1)	1.45 (1.35, 1.57)	<0.001	1.25 (1.14, 1.37)	<0.001
Q2–3 (0.76–<1.13)	6088	1985 (37.5)	ref		ref	
Q4 (≥1.13)	3045	1307 (54.9)	1.48 (1.38, 1.58)	<0.001	1.36 (1.25, 1.47)	<0.001

* Adjusted for sex, age, body mass index, survey year, regions, SBP, DBP, alcohol drinking, smoking, urban or rural residents, education levels, occupations, physical activity, intakes of energy, sodium and potassium. **^†^** Incidence rate of hypertension was calculated as the number of new-onset cases of hypertension divided by person-years of follow-up and was expressed per 1000 person-years.

**Table 4:** Association between dietary thiamine intake and risks of different components of new-onset hypertension.

Thiamine Intake, mg/day	N	Cases (Incidence Rate ^†^)	Crude Model		Adjusted Model *	
			HR (95% CI)	*p* Value	HR (95% CI)	*p* Value
Physician-diagnosed hypertension						
Quartiles						
Q1 (<0.76)	3024	200 (10.9)	ref		ref	
Q2 (0.76–<0.93)	3024	201 (7.9)	0.73 (0.60, 0.89)	0.002	0.98 (0.78, 1.22)	0.852
Q3 (0.93–<1.13)	3024	187 (6.9)	0.64 (0.52, 0.78)	<0.001	0.95 (0.74, 1.21)	0.669
Q4 (≥1.13)	3024	238 (10.1)	0.93 (0.77, 1.12)	0.459	1.30 (0.98, 1.71)	0.067
Categories						
Q1 (<0.76)	3024	200 (10.9)	1.47 (1.24, 174)	<0.001	1.03 (0.84, 1.27)	0.751
Q2–3 (0.76–<1.13)	6048	388 (7.4)	ref		ref	
Q4 (≥1.13)	3024	238 (10.1)	1.37 (1.16, 1.61)	<0.001	1.35 (1.11, 1.63)	0.002
Antihypertensive treatment during follow-up						
Quartiles						
Q1 (<0.76)	3026	132 (7.2)	ref		ref	
Q2 (0.76–<0.93)	3025	123 (4.8)	0.69 (0.54, 0.88)	0.003	1.02 (0.77, 1.35)	0.879
Q3 (0.93–<1.13)	3025	116 (4.3)	0.61 (0.47, 0.78)	<0.001	1.07 (0.79, 1.44)	0.685
Q4 (≥1.13)	3026	155 (6.5)	0.93 (0.74, 1.18)	0.564	1.56 (1.11, 2.19)	0.011
Categories						
Q1 (<0.76)	3026	132 (7.2)	1.55 (1.25, 1.92)	<0.001	0.96 (0.74, 1.25)	0.774
Q2–3 (0.76–<1.13)	6050	239 (4.5)	ref		ref	
Q4 (≥1.13)	3026	155 (6.5)	1.44 (1.18, 1.77)	<0.001	1.49 (1.17, 1.89)	0.001
New-onset SBP ≥140 mmHg or DBP ≥90 mmHg						
Quartiles						
Q1 (<0.76)	3044	886 (48.1)	ref		ref	
Q2 (0.76–<0.93)	3044	895 (34.9)	0.71 (0.64, 0.78)	<0.001	0.81 (0.73, 0.90)	<0.001
Q3 (0.93–<1.13)	3044	931 (34.1)	0.69 (0.63, 0.76)	<0.001	0.79 (0.70, 0.88)	<0.001
Q4 (≥1.13)	3045	1211 (50.9)	1.04 (0.95, 1.13)	0.411	1.09 (0.96, 1.24)	0.201
Categories						
Q1 (<0.76)	3044	886 (48.1)	1.44 (1.32, 1.56)	<0.001	1.25 (1.13, 1.37)	<0.001
Q2–3 (0.76–<1.13)	6088	1826 (34.5)	ref		ref	
Q4 (≥1.13)	3045	1211 (50.9)	1.49 (1.38, 1.60)	<0.001	1.36 (1.25, 1.49)	<0.001

* Adjusted for sex, age, body mass index, survey year, regions, SBP, DBP, alcohol drinking, smoking, urban or rural residents, education levels, occupations, physical activity, intakes of energy, sodium and potassium. ^†^ Incidence rate of hypertension was calculated as the number of new-onset cases of hypertension divided by person-years of follow-up and was expressed per 1000 person-years.

## Data Availability

The analytic code will be made available from the corresponding authors on request.

## Supplementary Materials

The following supporting information can be downloaded at: https://www.mdpi.com/article/10.3390/nu14163251/s1, Figure S1: Flow chart of study participants; Figure S2: Relation of dietary thiamine intake with risk of new-onset hypertension by sex based on restricted cubic splines; Table S1: The relationship of dietary thiamine intake with risk of new-onset hypertension, with further adjustment for intake of riboflavin, niacin, vitamin A, copper and zinc; Table S2: The relationship of dietary thiamine intake with risk of new-onset hypertension, with further adjustment for nuts, whole grains, refined grain, vegetables and fruits consumption.
