# Supplementary material for: U-Shaped Relation of Dietary Thiamine Intake and New-Onset Hypertension
Source: Nutrients. 2022 Aug 9;14(16):3251. doi: 10.3390/nu14163251 (PMC9415122; doi:10.3390/nu14163251)
Supplement: Supplementary file 1 [file nutrients-14-03251-s001.zip › nutrients-1808936-supplementary.pdf]

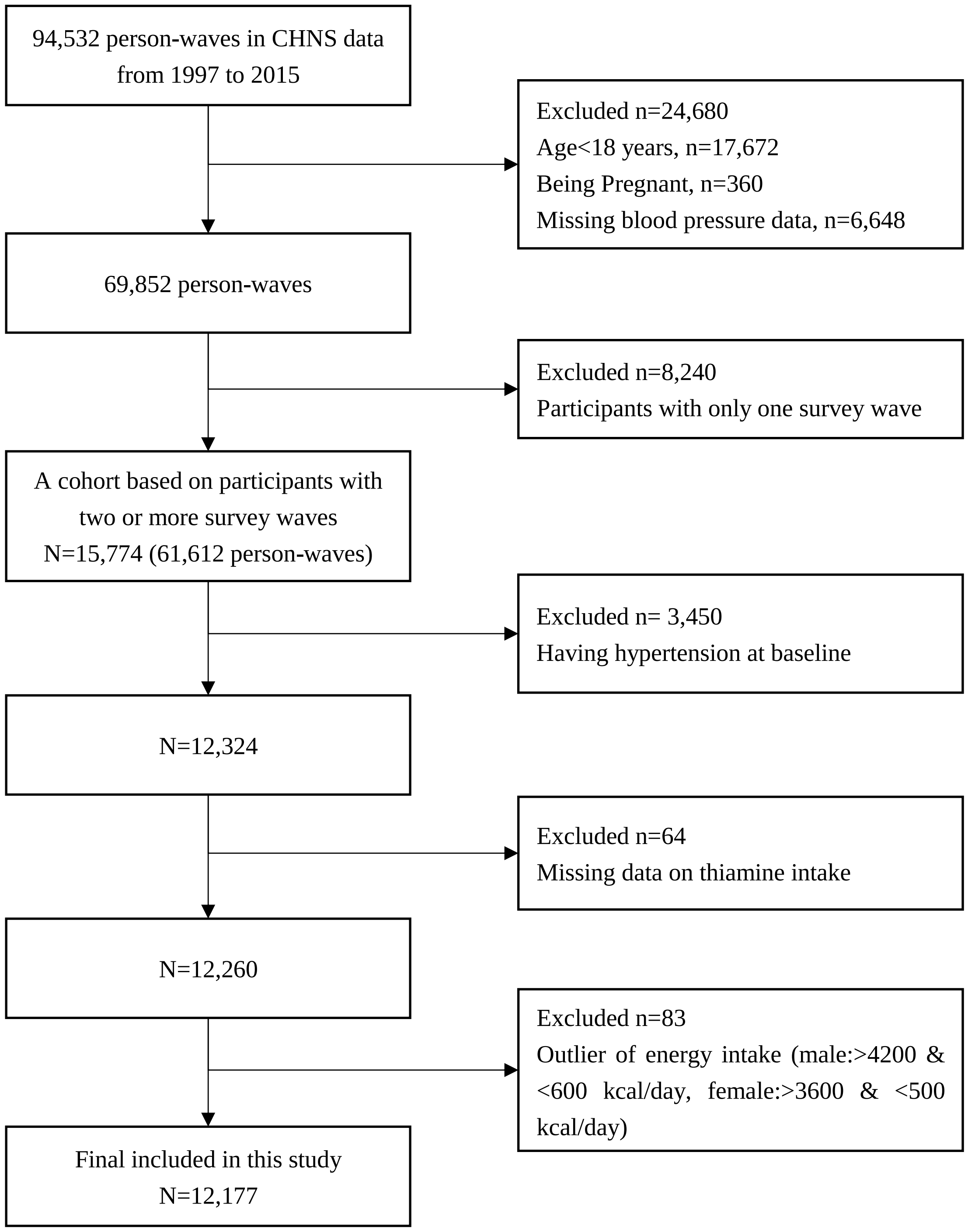

Supplemental Figure S1. Flow chart of study participants

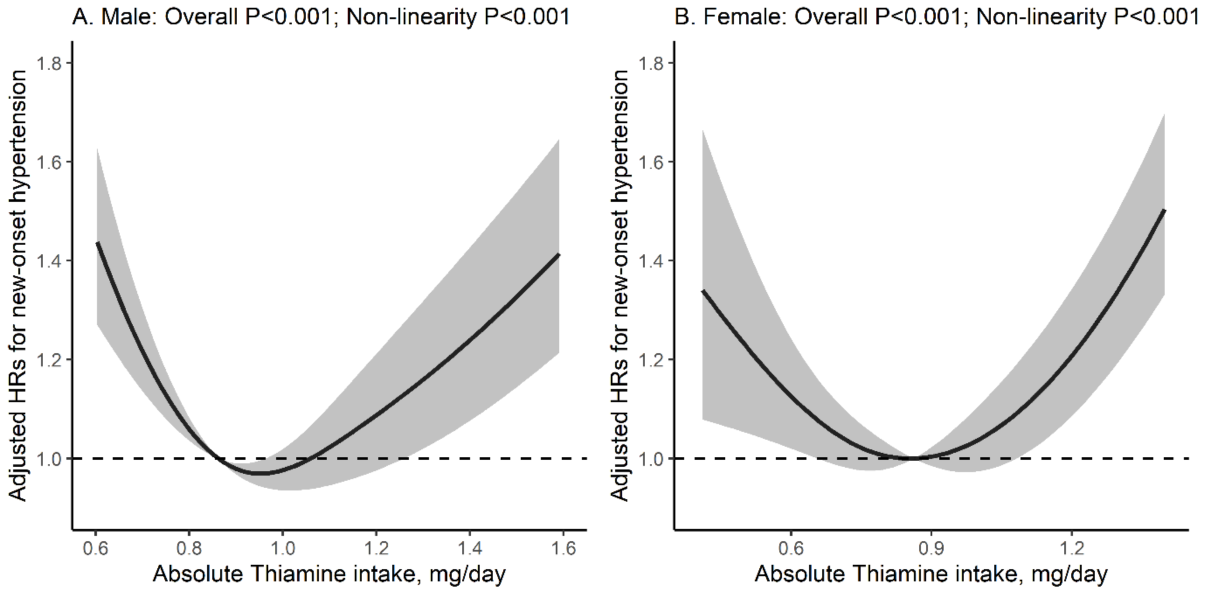

Supplemental Figure S2. Relation of dietary thiamine intake with risk of new-onset hypertension by sex based on restricted cubic splines

Adjusted for survey year, age, body mass index, systolic blood pressure, diastolic blood pressure, smoking, alcohol drinking, urban or rural residence, region, education, occupation, physical activity, intakes of energy, sodium and potassium.

Supplemental Table S1. The relationship of dietary thiamine intake with risk of new-onset hypertension, with further adjustment for intake of riboflavin, niacin, vitamin A, copper and zinc

Thiamine intake, mg/day	N	Cases (incidence rate†)	Crude model		Adjusted model*	
			HR (95%CI)	*P* value	HR (95%CI)	*P* value
Quartiles						
Q1 (<0.76)	3044	977 (53.1)	ref		ref	
Q2 (0.76-<0.93)	3044	981 (38.3)	0.70 (0.64,0.77)	<0.001	0.80 (0.73,0.89)	<0.001
Q3 (0.93-<1.13)	3044	1004 (36.8)	0.67 (0.62,0.74)	<0.001	0.75 (0.67,0.84)	<0.001
Q4 (≥1.13)	3045	1307 (54.9)	1.02 (0.94,1.10)	0.702	0.97 (0.85,1.09)	0.597
Categories						
Q1 (<0.76)	3044	977 (53.1)	1.45 (1.35,1.57)	<0.001	1.28 (1.17,1.40)	<0.001
Q2-3 (0.76-<1.13)	6088	1985 (37.5)	ref		Ref	
Q4 (≥1.13)	3045	1307 (54.9)	1.48 (1.38,1.58)	<0.001	1.26 (1.16,1.37)	<0.001

*Adjusted for survey year, age, sex, body mass index, systolic blood pressure, diastolic blood pressure, smoking, alcohol drinking, urban or rural residence, region, education, occupation, physical activity, intakes of energy, sodium and potassium, as well as intake of riboflavin, niacin, vitamin A, copper and zinc.

†Incidence rates per 1000 person years.

Supplemental Table S2. The relationship of dietary thiamine intake with risk of new-onset hypertension, with further adjustment for nuts, whole grains, refined grain, vegetables and fruits consumption

Thiamine intake, mg/day	N	Cases (incidence rate[†])	Crude model		Adjusted model[*]	
			HR (95%CI)	*P* value	HR (95%CI)	*P* value
Quartiles						
Q1 (<0.76)	3044	977 (53.1)	ref		ref	
Q2 (0.76-<0.93)	3044	981 (38.3)	0.70 (0.64,0.77)	<0.001	0.85 (0.77,0.94)	0.002
Q3 (0.93-<1.13)	3044	1004 (36.8)	0.67 (0.62,0.74)	<0.001	0.82 (0.74,0.91)	<0.001
Q4 (≥1.13)	3045	1307 (54.9)	1.02 (0.94,1.10)	0.702	1.07 (0.95,1.21)	0.297
Categories						
Q1 (<0.76)	3044	977 (53.1)	1.45 (1.35,1.57)	<0.001	1.19 (1.09,1.31)	<0.001
Q2-3 (0.76-<1.13)	6088	1985 (37.5)	ref		Ref	
Q4 (≥1.13)	3045	1307 (54.9)	1.48 (1.38,1.58)	<0.001	1.29 (1.18,1.40)	<0.001

[*] Adjusted for age, sex, body mass index, survey year, SBP, DBP, smoking, alcohol drinking, urban or rural residents, regions, education levels, occupations, physical activity levels, intakes of energy, sodium and potassium, as well as nuts, whole grains, refined grain, vegetables and fruits consumption.

[†]Incidence rates per 1000 person years.
